# Longitudinal association between preschool fussy eating and body composition at 6 years of age: The Generation R Study

**DOI:** 10.1186/s12966-015-0313-2

**Published:** 2015-12-14

**Authors:** Lisanne M. de Barse, Henning Tiemeier, Elisabeth T. M. Leermakers, Trudy Voortman, Vincent W. V. Jaddoe, Lisa R. Edelson, Oscar H. Franco, Pauline W. Jansen

**Affiliations:** The Generation R Study Group, Erasmus MC-University Medical Center, PO Box 2040, 3000 CA Rotterdam, The Netherlands; Department of Epidemiology, Erasmus MC-University Medical Center, PO Box 2040, 3000 CA Rotterdam, The Netherlands; Department of Child and Adolescent Psychiatry/Psychology, Erasmus MC-University Medical Center, PO Box 2060, 3000 CB Rotterdam, The Netherlands; Department of Psychiatry, Erasmus MC-University Medical Center, PO Box 2040, 3000 CA Rotterdam, The Netherlands; Department of Pediatrics, Erasmus MC-University Medical Center, PO Box 2060, 3000 CB Rotterdam, The Netherlands; Taste and Behavioral Sciences, Nestlé Research Center, Lausanne, Switzerland; Institute of Psychology, Erasmus University Rotterdam, PO Box 1738, 3000 DR Rotterdam, The Netherlands

**Keywords:** Fussy eating, Body composition, Underweight, Children, Epidemiology, Cohort

## Abstract

**Background:**

Children’s fussy eating behavior has been related to both underweight and overweight in cross-sectional studies, but the direction of these associations and the relation with more detailed measures of body composition remains unclear. We aimed to examine whether fussy eating at age 4 years is longitudinally related to body mass index (BMI), fat mass index (FMI) and fat-free mass index (FFMI) at 6 years of age.

**Methods:**

This study was embedded in Generation R, a population-based, prospective cohort. Data were available for 4191 children. The Children’s Eating Behaviour Questionnaire (CEBQ), administered at age 4 years, was used to derive a fussy eating profile. This profile is characterized by high scores on food avoidant scales and low scores on food approach scales. At age 6 years, height and weight were measured at our research center. Body fat and fat-free mass were measured using Dual-energy-X-ray absorptiometry. We used age- and sex-specific standard deviation scores (SDS) for all outcomes.

**Results:**

After adjustment for confounders, the fussy eating profile was related to lower BMI-SDS (B = −0.37, 95 % CI: −0.47;−0.26), lower FMI-SDS (B = −0.22, 95 % CI: −0.33;−0.12) and lower FFMI-SDS (B = −0.41, 95 % CI: −0.54;−0.29). When adjusting for baseline BMI at 4 years, the fussy eating profile predicted a 0.11 lower BMI-SDS at age 6 (95 % CI: −0.19;−0.04). This change in BMI was mainly due to a decrease in FFMI (B = −0.19, 95 % CI: −0.29;−0.09). Fussy eaters also had a higher risk of becoming underweight than non-fussy eaters (OR = 2.28, 95 % CI: 1.34;3.87).

**Conclusions:**

Our findings suggest that young fussy eaters are at risk of having a lower fat free mass and of becoming underweight over a 2-year period. This implies that fussy eaters may benefit from careful monitoring to prevent an adverse growth development.

**Electronic supplementary material:**

The online version of this article (doi:10.1186/s12966-015-0313-2) contains supplementary material, which is available to authorized users.

## Background

Although obesogenic eating behaviors and weight development in childhood have been widely studied [1], fussy eating is, despite its high prevalence, a surprisingly unexplored area. Fussy eating – also called ‘picky’, ‘selective’, or ‘choosy’ eating – is a common phenomenon in preschool-aged children, with prevalence estimates ranging from 14 to 50 % [2, 3]. Fussy eaters often reject new food items (food neophobia), but they are particularly characterized by their consistent rejection of specific familiar foods [4], especially vegetables [5, 6]. This consistent refusal of specific food items may result in a restricted dietary variety [4], which could have consequences for a child’s health, growth, and development. It is possible that fussy eaters have an insufficient energy intake [5], resulting in underweight. However, fussy eaters may compensate for their limited intake of vegetables and other disliked foods by eating more palatable, energy-dense foods, giving them a relatively high energy intake. Indeed, in a study of 8- to 12-year-old children, fussy eaters had a higher preference for fast food than did non-fussy eaters [7], suggesting that they may be at risk of overweight. Both overweight and underweight in childhood have been related to several adverse health outcomes [8–11]. Obese children are more likely to face emotional and social problems [8, 9], and they are at risk of cardiovascular health problems across the life course [8]. Although less pronounced, child underweight is also related to increased psychosocial problems, a poorer quality of life [10] and physical health problems like a relatively low bone mass [11] which may put these children at greater risk of fractures [12].

Several studies have reported that fussy eating in children is associated with a lower body mass index (BMI) [6, 13–15], lower body fat percentage [6], and underweight [3, 13, 16, 17]. However, other studies did not find any association between children’s fussy eating and weight status [18–23], while Finistrella et al. reported that overweight/obese children were more likely to be fussy, neophobic eaters than normal weight children [24]. Except for two studies [6, 23], research has focused on BMI only, and not on other measures of body composition. Distinguishing fat mass from lean mass, however, provides a better insight into children’s overall body composition. For example, fussy eaters may have a normal or even low overall body weight, potentially masking relatively high levels of body fat due to a high intake of energy-dense food [7]. Another limitation of most previous studies is their cross-sectional design, in which it is difficult to make causal inferences and therefore, longitudinal research is needed.

In the current study, we aimed to examine the longitudinal association between children’s fussy eating at the age of 4 years and body mass at 6 years of age. Further, we aimed to explore whether fussy eaters differ from non-fussy eaters in height and with respect to their fat mass and lean mass at 6 years. This knowledge about fussy eating in childhood and its impact on weight development and body composition will inform whether preventative intervention strategies are needed for fussy eaters.

## Methods

### Study design & procedure

This study was embedded in the Generation R Study, a population-based cohort from fetal life onwards [25]. The Generation R Study was designed to identify early biological, environmental, and social determinants of growth, development, and health. Pregnant women living in Rotterdam, The Netherlands, with an expected delivery date between April 2002 and January 2006 were invited to participate. Assessments included physical examinations and parental questionnaires. Written informed consent was obtained from all participating parents and the local Medical Ethical Committee has approved this study. Further information about the study is available elsewhere [25].

### Participants

Full consent for the postnatal phase of the Generation R Study was obtained for 7295 children and their parents. Of these, 4914 children (67 %) had available information on their eating behavior. In 4191 children (85.3 %), information on height and weight was available at follow up. Of those, a Dual-energy-X-ray absorptiometry scan was missing in 126 children. Therefore, the population for analysis was 4191 for analyses with weight-related outcomes and 4065 for analyses with fat and fat-free mass as outcomes.

### Measures

#### Children’s fussy eating

Eating behavior was assessed with the Children’s Eating Behaviour Questionnaire (CEBQ) at 4 years of age [26]. The CEBQ is a validated, multi-dimensional parent-report questionnaire designed to measure differences in children’s eating behaviors. The CEBQ consists of eight scales, each containing three to six items (in total 35 items). Parents rated the frequency of their children’s eating behavior on a Likert scale from 1 (never) to 5 (always). Scale scores were calculated by summing the items if at least 75 % were completed. Scale scores were corrected for the number of completed items. Research has shown that the CEBQ has good psychometric properties in terms of internal reliability, test-retest reliability, and factor structure [19, 26].

In this study, we used a fussy eating profile, based on a previously performed latent profile analysis on five CEBQ subscales [27]. Children assigned to the fussy eating profile were characterized by low scores on food responsiveness and enjoyment of food (food approach behaviors) and high scores on satiety responsiveness, food fussiness, and slowness in eating (food avoidance behaviors) [27]. In our analyses, fussy eaters were compared with non-fussy eaters, i.e. all children who were assigned to another eating profile (avoidant, moderate, responsive, joyful, or approaching eating profile).

For sensitivity analyses, we also included fussy eating trajectories based on the Child Behavior Checklist [28] (CBCL) assessed at 1.5, 3 and 6 years of age, as previously described in detail [29]. The previously created fussy eating trajectories [29] were used: 1) never fussy eaters, 2) ‘remitting’ fussy eaters: fussy eater at 1.5 year and/or at 3 years, but not at 6 years; 3) late-onset fussy eaters: only fussy eater at 6 years; 4) persistent fussy eaters: fussy eater at all assessment waves (1.5, 3 and 6 years).

#### Children’s body composition

Children visited our research center at age 6 years, where trained staff performed several measurements of body composition. Height was measured in standing position using a Harpenden stadiometer and weight was measured without heavy clothing using a mechanical personal scale. Height and weight were used to calculate body mass index (BMI, kg/m^2^). Age- and sex-adjusted standard deviation (SD) scores for height and BMI were calculated using Dutch reference growth curves [30]. Children were classified into underweight, normal weight, overweight, or obese, using international age- and sex-specific cut-offs [31, 32]. Body fat mass, bone mass, and lean mass were measured by Dual-energy-X-ray absorptiometry (DXA) scans (iDXA, GE-Lunar, 2008, Madison, WI, USA), using enCORE software v.13.6. Fat mass index (FMI) was calculated as total fat mass (kg) divided by squared height (m^2^). Likewise, children’s fat-*free* mass index (FFMI) was calculated ((sum of bone and lean mass in kg)/height in m^2^). Age- and sex-adjusted standard deviation (SD) scores for FMI and FFMI were calculated with the residual method in all participating Generation R children who had available data on FMI or FFMI (*N* = 6491).

#### Covariates

Several maternal and child characteristics that may confound the association between children’s fussy eating and body composition were considered. During pregnancy, a questionnaire was used to assess *maternal age* (in years). In the same questionnaire, *maternal psychiatric symptoms* were assessed with the Brief Symptom Inventory (overall mean score, range: 0–4), a 53-item, validated self-report questionnaire reflecting a diverse spectrum of psychiatric problems [33, 34]. *Birth weight* and *child sex* were obtained from medical records completed by midwives and gynecologists. In postnatal questionnaires, *breastfeeding duration* (in months), *age at introduction of fruit and vegetables* (in months)*, history of any food allergy at age 1 year* (yes/no), *maternal educational level*, monthly *family income*, *child ethnicity* (based on country of birth of both parents), and *children’s functional constipation in the year before the fourth birthday* (based on the Rome II criteria) [35] were assessed. Maternal height and weight were measured by trained staff at the research center (when children were 6 years of age) and were used to calculate *maternal BMI* (kg/m^2^).

To enhance insight into the directionality of the associations, we accounted for BMI at baseline (age 4 years). Children visited the municipal Child Health Centers around their fourth birthday. Height and weight were measured by trained staff as part of a routine health care program. Similar to anthropometrics at age 6 years, BMI SD scores for age and sex were calculated using Dutch reference scores [30].

### Statistical analyses

To determine whether fussy eaters differed in their body composition from non-fussy eaters, we performed three separate linear regression analyses with fussy eating profile (fussy eaters vs. non-fussy eaters) as the exposure and BMI-SDS, FMI-SDS, and FFMI-SDS as outcomes. Next, we performed a multinomial logistic regression analysis to assess whether fussy eaters had a higher risk of being underweight, overweight, or obese (reference group: normal weight) than non-fussy eaters. All analyses were adjusted for potential confounders that changed the effect estimates by 5 % or more [36] (only food allergies did not reach this criterion and was thus left out of the analyses). In separate models, we adjusted the analyses for children’s BMI-SDS at baseline (age 4 years) to assess whether fussy eating behavior at 4 years predicted change in body composition measures at age 6 years.

Several sensitivity analyses were performed. First, the associations of the different fussy eating trajectories from ages 1,5 to 6 years with BMI-SDS, FMI-SDS, and FFMI-SDS were examined with linear regression analyses, controlling for potential confounders and baseline BMI. Second, we checked the association between children’s fussy eating profile and height (age- and sex-specific SD-scores), adjusting for potential confounders. In a separate model, we adjusted this analysis for children’s height at baseline.

Missing values on covariates were estimated using multiple imputation techniques [37]. All statistical analyses were performed with SPSS 20.1.

## Results

### Population characteristics

Of all included children, 5.7 % were assigned to the fussy eating profile (Table 1). Most children had a Dutch ethnic background (66.0 %) and had mothers with a relatively high education (higher vocational training or academic degree, 63.7 %).

**Table 1:** General characteristics of 4191 children included in the study

Population characteristics		*N*	Percentage, mean (SD), or median (IQR)^a^
Child sex	% Boy	2085	49.7
Birth weight (mean grams)		4189	3441 (565)
Child ethnicity	% Dutch	2768	66.0
	% Moroccan	136	3.2
	% Surinamese & Dutch Antillean	305	7.3
	% Turkish	243	5.8
	% Other, Western (mainly European)	394	9.4
	% Other, non-Western	345	8.2
Breastfeeding duration (median months)		4191	3.5 (7.0)
Introduction of fruits and vegetables	% <3 months	265	6.3
	% 3–6 months	3477	83.0
	% >6 months	449	10.7
Functional constipation at age 4 years	% Yes^b^	616	14.7
Fussy eating profile	% Fussy eater	240	5.7
Child BMI age 4 years (mean SDS)		4191	0.1 (0.9)
Child BMI age 6 years (mean SDS)		4191	0.2 (0.9)
Maternal age (mean years)		4191	31.6 (4.6)
Maternal educational level	% Low^c^	374	8.9
	% Medium^c^	1148	27.4
	% High^c^	2669	63.7
Family income per month (median in €)		4191	3600 (2600)
Maternal BMI (median)		4191	24.1 (5.6)
Maternal psychiatric symptoms (median score)		4191	0.1 (0.2)

^a^Values are percentages for categorical variables, means (standard deviation) for continuous normally distributed variables and median (interquartile range) for continuous non-normally distributed variables, derived from the imputed dataset. ^b^Children who had had less than two bowel movements per week or predominantly hard feces for at least two successive weeks were classified as functional constipated. ^c^Low education: ranging from no education up to high school level, medium: lower vocational training, high: higher vocational education and higher academic education

### Fussy eating and body composition

The associations between the fussy eating profile and body composition measures are presented in Table 2 (see also Additional file 1: Table S1 for the unadjusted associations). Children classified as fussy eaters had a 0.37 lower BMI SD-score at age 6 years than other children (95 % CI: −0.47; −0.26). Fussy eaters also had a 0.22 lower FMI-SDS (95 % CI: −0.33; −0.12) and a 0.41 lower FFMI-SDS (95 % CI: −0.54; −0.29). After adjustment for BMI at age 4 years, the effect estimates attenuated, but fussy eating remained significantly associated with a lower BMI SD-score (B = −0.11, 95 % CI: −0.19; −0.04) and with a lower FFMI-SDS at age 6 years (B = −0.19, 95 % CI: −0.29; −0.09). Similarly, sensitivity analyses with the fussy trajectories showed that persistent fussy eaters – but not remittent or late onset fussy eaters – had a lower BMI and FFMI after correcting for baseline BMI (Additional file 1: Table S2).

**Table 2:** Child fussy eater profile at 4 years of age and body composition at 4 and 6 years of age

	Body composition at 4 years B (95 % CI)^a^	Body composition at 6 years B (95 % CI)^a^		
Fussy eating profile at 4 years	Body mass index-SDS *N* = 4191	Body mass index –SDS *N* = 4191	Fat mass index-SDS *N* = 4065	Fat-free mass index-SDS *N* = 4065
Model 1: adjusted for potential confounders^b^				
Fussy eater profile vs non-fussy eater profile	−0.38 (−0.50; −0.25)***	−0.37 (−0.47; −0.26)***	−0.22 (−0.33; −0.12)***	−0.41 (−0.54; −0.29)***
Model 2: additionally adjusted for BMI at age 4^c^				
Fussy eater profile vs non-fussy eater profile	–	−0.11 (−0.19; −0.04)**	−0.02 (−0.11; 0.07)	−0.19 (−0.29; −0.09)***

^a^Values are regression coefficients (95 % confidence intervals). **p <0.01, ***p <0.001. All body composition outcomes are age- and sex- adjusted standard deviation scores. ^b^Model 1: adjusted for potential confounders: maternal age, educational level, BMI, and psychiatric symptoms during pregnancy; family income; child ethnicity, sex, age when CEBQ was filled out, birth weight, and functional constipation at age 4 years; breastfeeding, and introduction of fruit and vegetables. ^c^Model 2: model 1 + additionally adjusted for children’s BMI at age 4 years

Additional sensitivity analyses revealed that fussy eaters were also shorter than non-fussy eaters (adjusted B = −0.26, 95 % CI: −0.39; −0.14), but this effect estimate attenuated towards null when we adjusted for height at age 4 years (data not shown), implying that fussy eating did not predict less height growth over this 2-year period.

### Fussy eating and the risk of underweight, overweight, or obesity

**Fig. 1:**
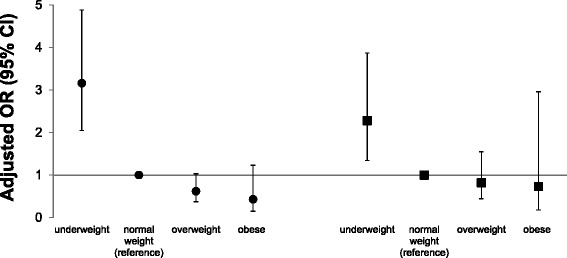
Child fussy eater profile at age 4 years and risk of being underweight, overweight, or obese at 6 years. ● adjusted for potential confounders. ■ additionally adjusted for baseline BMI at 4 years

Fussy eaters had a greater risk of being underweight at age 6 years than non-fussy eaters, even after adjustment for baseline BMI at 4 years (OR = 2.28, 95 % CI: 1.34; 3.87) (Fig. 1 and Additional file 1: Table S3). Fussy eaters were not at risk of being overweight or obese.

## Discussion

Using a longitudinal design within a large population-based cohort, fussy eating at 4 years of age predicted a lower BMI at age 6. In addition, preschoolers with a fussy eating pattern were at risk of developing underweight over 2 years’ time. The analyses disentangling the different components of body composition indicated that the lower BMI of fussy eaters is mainly explained by a lower fat free mass.

The finding that fussy eating was associated with underweight in children is in line with previous studies that also found a lower body fat percentage [6] and lower weight status among fussy eaters [3, 6, 13–16]. However, several other studies did not find any association with weight status [18–23] and some even reported that fussy eating was related to overweight [24, 38]. This discrepancy in findings might indicate that fussy eaters are a heterogeneous group of children who differ, for instance, in the severity of their food fussiness. Some children may exhibit “severe” fussy eating behavior, either in terms of chronicity or the nature of their behavior. The latter might be applicable for our group of fussy eaters, as they not only scored high on the CEBQ’s food fussiness scale, but also on other avoidant eating behaviors: they got full more easily, were slower eaters, and did not enjoy eating as much as other children [27]. Possibly, the combination of these behaviors reflects a more severe fussy eating pattern that may result in underweight. The low prevalence of this fussy eating profile in our sample (5.7 %) underlines that these children form a distinct group of fussy eaters. Further support for this reasoning comes from our analyses with the fussy eating trajectories and from another longitudinal study [3] both showing that only *persistent* fussy eaters, which could be perceived as severe fussy eaters in terms of chronicity, were at risk of a lower BMI and underweight. In contrast, children who were depicted as fussy eaters at only one or two time points had a rather normal weight development. This fits a broader developmental perspective that a period of fussy eating during toddlerhood can be a normal developmental phase, not necessarily warranting clinical attention. However, clinicians and parents should pay attention to severe forms of fussy eating behavior as it may have consequences for weight development, or it could be an indicator of further developmental problems, as fussy eating and sensory sensitivity are also often prevalent in children with pervasive developmental disorders [39].

In addition to the existing literature that comprises mostly cross-sectional research, our study adds to the discussion about the direction of the association between fussy eating and body composition. Due to the cross-sectional design of most previous studies [3, 6, 13, 14, 19, 20], it was debated whether children’s fussy eating behaviors influence weight status or the reverse. In response to this discussion, Jaarsveld, Llewelyn, Johnson and Wardle [40] were among the first to test bidirectional associations between eating behaviors that are captured in our fussy eating profile (e.g. slowness in eating, satiety responsiveness) and weight in infancy. They concluded that the pathway of eating behaviors influencing weight was the strongest, though the reverse pathway from weight to eating behaviors was not completely absent. Although we could not test bidirectional associations, the availability of repeated measurements of child BMI enabled us to adjust the analyses for BMI at baseline. Consistent with the study of Jaarsveld et al., [40] our results also suggest that fussy eating at age 4 predicts a significant decrease in children’s BMI and more specifically fat-free mass over the next 2 years. However, from the current study, we cannot make inferences about the directionality of the association in the first 4 years of life. Fussy eaters had a lower BMI at baseline (age 4 years), so it is possible that children’s weight in infancy or toddlerhood may already have influenced their eating behavior, for instance through effects on parental feeding practices. Jansen et al. previously showed that a relatively low BMI in children elicited pressuring feeding behaviors in parents [41]. While parents may intend to increase their children’s food intake, pressure to eat could have the opposite effect. Pressuring feeding strategies may induce negative reactions in children towards foods [42], thereby exacerbating or contributing to the development of fussy eating. Future studies should explore the pathway from body composition to fussy eating, using repeated measurements and taking parents’ feeding strategies into account as a possible mediator.

Fussy eating was related to different aspects of body composition. Although we carefully adjusted the analyses for numerous potential confounding factors, there is still a possibility that the association is flawed by residual confounding, for instance due to potential measurement error or misclassification of our confounders [43]. However, assuming that our findings represent at least partly a true association, fussy eating seems to have an overall effect on growth. The strong association of fussy eating with a lower fat-free mass suggests that fussy eating is associated with a lower muscle mass which is worrisome given that a higher muscle mass and muscle strength are considered to have a beneficial effect on metabolic and cardiovascular health [44–46]. The potential adverse effects of fussy eating on health are also underlined by our finding that fussy eaters were at risk of developing underweight over time. We hypothesize that this overall effect on growth development is due to an insufficient energy intake or a relatively poor diet quality among fussy eaters. Unfortunately, we were not able to test this hypothesis as we lacked data on food intake between 4 and 6 years of age. However, a poor overall diet quality is likely [47] given the restricted diet variety that fussy eaters have [4].

The current study was strengthened by its population-based, prospective design with multiple assessments of BMI, which allowed us to examine the longitudinal associations between fussy eating and body composition. Another strength was the inclusion of detailed body composition measures. While most previous studies focused on body mass only, we distinguished fat mass from fat-free mass. One of the studies that also included fat and fat-free mass used skinfold thickness [23] which is a less reliable method than the DXA measurements that we used [48]. Ideally, multiple DXA measurements would have been able to account for baseline fat and fat-free mass. We also lacked information on concurrent food intake of children which would be interesting to assess in future studies as a potential mediating factor in the association between fussiness and body composition. Another possible limitation is that fussy eating was measured by parent report, and parents of smaller children might be more likely to perceive their children to be fussy eaters. However, we addressed this by controlling for baseline BMI at 4 years. Finally, as with all cohort studies, selective follow up is a potential limitation. In the Generation R Study, loss to follow up is higher in those from low socioeconomic status and non-Western origin [25], which to some extent, limits generalizability of the results to the general population.

## Conclusions

Although fussy eating could be considered as a normal phase of development [29, 39], our findings highlight the possible adverse effect of more severe fussiness on healthy growth. Young fussy eaters are at risk of developing underweight and a relatively low fat-free mass. Health care practitioners should carefully monitor fussy eaters and their dietary quality, particularly children who not only reject certain types of foods, but also show a pattern of slow, joyless eating of foods. In their advice to parents, it is important that health care practitioners show understanding for parents’ concerns, frustrations, and possible adverse – but imaginable - reactions to their child’s food fussiness. Although coercive and pressuring feeding strategies are understandable, the possible counterproductive effects should be explained (i.e. more food refusal, negative atmosphere) [49, 50]. There is no golden standard for overcoming fussy eating yet, but research suggests that repeated exposure to a diversity of food items without coercion of eating is key for food acceptance [49]. Moreover, it is advised to encourage parents to cook [51] and eat [49] together with their children, so that parents act as a role model and children can imitate their parents’ eating behaviors [49]. Although these strategies may improve food intake [51] and food enjoyment [50] of children, more research is needed to study the effectiveness for such interventions in fussy eaters. Future studies should also follow fussy eaters over a longer period of time to examine whether fussy eating also has long-term adverse consequences for body composition development and related health outcomes.

## Additional file

Additional file 1:
**Tables S1, S2 and S3.** The unadjusted associations between the fussy eating profile and child body composition are presented in Table S1. Sensitivity analyses with the fussy eating trajectories and child body composition are presented in Table S2. Table S3. shows the results on which Fig. 1 is based. (DOCX 17 kb)

## Abbreviations

BMI: body mass index
CEBQ: Children’s Eating Behaviour Questionnaire
CI: confidence interval
DXA: Dual-energy-X-ray absorptiometry
IQR: interquartile range
FFMI: fat-free mass index
FMI: fat mass index
SD: standard deviation
SDS: standard deviation score
