# Supplementary material for: Longitudinal association between preschool fussy eating and body composition at 6 years of age: The Generation R Study
Source: Int J Behav Nutr Phys Act. 2015 Dec 14;12:153. doi: 10.1186/s12966-015-0313-2 (PMC4678491; doi:10.1186/s12966-015-0313-2)
Supplement: Additional file 1: — Tables S1, S2 and S3. The unadjusted associations between the fussy eating profile and child body composition are presented in Table S1. Sensitivity analyses with the fussy eating trajectories and child body composition are presented in Table S2. Table S3. shows the results on which Fig. 1 is based. (DOCX 17 kb) [file 12966_2015_313_MOESM1_ESM.docx]

Additional file

*Manuscript: Longitudinal association between preschool fussy eating and body composition at 6 years of age: The Generation R Study*

Lisanne M. de Barse, MSc, Henning Tiemeier, MD PhD, Elisabeth T.M. Leermakers, PhD, Trudy Voortman, PhD, Vincent V.W. Jaddoe, MD PhD, Lisa R. Edelson, PhD, Oscar H. Franco, MD PhD, Pauline W. Jansen, PhD

**Description of Additional file 1:**

The unadjusted associations between the fussy eating profile and child body composition are presented in Table S1.

Sensitivity analyses with the fussy eating trajectories and child body composition are presented in Table S2.

Table S3 shows the results on which Fig. 1 is based.

**Table S1. Unadjusted associations between child fussy eater profile and body composition**

|  | **Body composition at 4 years**  **B (95% CI)^a^** |  | **Body composition at 6 years**  **B (95% CI)^a^** | | |
| --- | --- | --- | --- | --- | --- |
| **Fussy eating profile at 4 years** | **Body mass index-SDS**  **N=4191** |  | **Body mass index –SDS N=4191** | **Fat mass index-SDS**  **N=4065** | **Fat-free mass index-SDS**  **N=4065** |
| **Model 0: unadjusted** |  |  |  |  |  |
| Fussy eater profile vs non-fussy eater profile | -0.37 (-0.50; -0.24)*** |  | -0.31 (-0.43; -0.20)*** | -0.13 (-0.24; -0.02)* | -0.45 (-0.57; -0.32)*** |

^a^Values are regression coefficients (95% confidence intervals). *p<0.05, ***p<0.001. All body composition outcomes are age- and sex- adjusted standard deviation scores.

**Table S2. Child fussy eating trajectories and body composition at 6 years of age**

|  | **Body composition at 6 years**  **B (95% CI)^a^** | | |
| --- | --- | --- | --- |
| **Fussy eating trajectories** | **Body mass Index –SDS N=3094** | **Fat mass index-SDS**  **N=3007** | **Fat-free mass index-SDS**  **N=3007** |
| **Model 1: adjusted for potential confounders^b^ and for baseline BMI^c^** |  |  |  |
| Never fussy eating | Reference | Reference | Reference |
| Remitting fussy eating (onset at 1.5 or 3 years, but not persisting) | -0.03 (-0.08; 0.01) | -0.04 (-0.09; 0.01) | -0.04 (-0.10; 0.02) |
| Late onset fussy eating (at 6 years) | -0.04 (-0.15; 0.07) | 0.00 (-0.12; 0.12) | -0.11 (-0.26; 0.04) |
| Persistent fussy eating (from 1.5 years till 6 years) | -0.39 (-0.51; -0.26)*** | -0.29 (-0.42; -0.16)*** | -0.32 (-0.47; -0.17)*** |

^a^Values are regression coefficients (95% confidence intervals). ***p<0.001. All body composition outcomes are age- and sex- adjusted standard deviation scores. ^b^Adjusted for potential confounders: maternal age, educational level, BMI, and psychiatric symptoms during pregnancy; family income; child ethnicity, sex, age when CEBQ was filled out, birth weight, and functional constipation at age 4 years; breastfeeding, and introduction of fruit and vegetables. ^c^Additionally adjusted for baseline BMI. For remitting fussy eating, we adjusted for baseline BMI at 1.3years and at 3 years. For late onset fussy eating, we adjusted for baseline BMI at 4 years. For persistent fussy eating, we adjusted for baseline BMI at 1.3years.

**Table S3. Child fussy eater profile and risk of being underweight, overweight, or obese**

|  | **OR (95% CI) for risk of being underweight, overweight, or obese^a^** | | | |
| --- | --- | --- | --- | --- |
|  | **Underweight**  **N=212 (5.1%)** | **Normal weight**  **N=3401 (81.3%)** | **Overweight**  **N=454 (10.9%)** | **Obese**  **N=116 (2.8%)** |
| **Model 0: unadjusted** |  |  |  |  |
| Fussy eater profile vs non-fussy eater profile | 2.98 (1.98; 4.48)*** | Reference | 0.76 (0.47; 1.23) | 0.62 (0.23; 1.70) |
| **Model 1: adjusted for potential confounders^b^** |  |  |  |  |
| Fussy eater profile vs non-fussy eater profile | 3.16 (2.05; 4.88)*** | Reference | 0.62 (0.37; 1.03) | 0.43 (0.15; 1.23) |
| **Model 2: additionally adjusted for BMI at age 4^c^** |  |  |  |  |
| Fussy eater profile vs non-fussy eater profile | 2.28 (1.34; 3.87)** | Reference | 0.82 (0.44; 1.55) | 0.73 (0.18; 2.96) |

^a^Values are odds ratios (95% confidence intervals). **p<0.01, ***p<0.001. ^b^Model 1: adjusted for potential confounders: maternal age, educational level, BMI, and psychiatric symptoms during pregnancy; family income; child ethnicity, sex, age when CEBQ was filled out, birth weight, and functional constipation at age 4 years; breastfeeding, and introduction of fruit and vegetables. ^c^Model 2: model 1 + additionally adjusted for children’s BMI at age 4 years.
